# The Product of Resting Heart Rate Times Blood Pressure Is Associated with High Brachial-Ankle Pulse Wave Velocity

**DOI:** 10.1371/journal.pone.0107852

**Published:** 2014-09-16

**Authors:** Anxin Wang, Jie Tao, Xiuhua Guo, Xuemei Liu, Yanxia Luo, Xiurong Liu, Zhe Huang, Shuohua Chen, Xingquan Zhao, Jost B. Jonas, Shouling Wu

**Affiliations:** 1 Department of Neurology, Beijing Tiantan Hospital, Capital Medical University, Beijing, China; 2 Department of Epidemiology and Health Statistics, School of Public Health, Capital Medical University, Beijing, China; 3 Beijing Municipal Key Laboratory of Clinical Epidemiology, Capital Medical University, Beijing, China; 4 Kailuan Hospital, Hebei United University, Tangshan, China; 5 Department of Ophthalmology, Medical Faculty Mannheim of the Ruprecht-Karls-University of Heidelberg, Heidelberg, Germany; Shanghai Institute of Hypertension, China

## Abstract

**Objective:**

To investigate potential associations between resting heart rate, blood pressure and the product of both, and the brachial-ankle pulse wave velocity (baPWV) as a maker of arterial stiffness.

**Methods:**

The community-based “Asymptomatic Polyvascular Abnormalities in Community (APAC) Study” examined asymptomatic polyvascular abnormalities in a general Chinese population and included participants with an age of 40+ years without history of stroke and coronary heart disease. Arterial stiffness was defined as baPWV≥1400 cm/s. We measured and calculated the product of resting heart rate and systolic blood pressure (RHR-SBP) and the product of resting heart rate and mean arterial pressure (RHR-MAP).

**Results:**

The study included 5153 participants with a mean age of 55.1±11.8 years. Mean baPWV was 1586±400 cm/s. Significant (*P*<0.0001) linear relationships were found between higher baPWV and higher resting heart rate or higher arterial blood pressure, with the highest baPWV observed in individuals from the highest quartiles of resting heart rate and blood pressure. After adjusting for confounding parameters such as age, sex, educational level, body mass index, fasting blood concentrations of glucose, blood lipids and high-sensitive C-reactive protein, smoking status and alcohol consumption, prevalence of arterial stiffness increased significantly (*P*<0.0001) with increasing RHR-SBP quartile (Odds Ratio (OR): 2.72;95%Confidence interval (CI):1.46,5.08) and increasing RHR-MAP (OR:2.10;95%CI:1.18,3.72). Similar results were obtained in multivariate linear regression analyses with baPWV as continuous variable.

**Conclusions:**

Higher baPWV as a marker of arterial stiffness was associated with a higher product of RHR-SBP and RHR-MAP in multivariate analysis. In addition to other vascular risk factors, higher resting heart rate in combination with higher blood pressure are risk factors for arterial stiffness.

## Introduction

Brachial-ankle pulse wave velocity (baPWV) is an index of arterial stiffness and a marker of atherosclerosis that can be measured noninvasively [1]–[4]. It has been shown to be an independent predictor of all-cause and cardiovascular mortality as well as an indicator for cardiovascular diseases [3], [5]–[7]. With the increasing use of baPWV in clinical practice as measure of arterial stiffness, the importance of understanding its associations with other cardiovascular factors has increased. Although the mechanisms responsible for arterial stiffening have not conclusively been explored yet, it has been discussed that arterial stiffness may be due to structural changes in the tunica media of the arteries. Based on mechanical considerations, one may postulate that these changes, including fatigue fracture of elastin [8], are correlated with the cyclic stress caused by the frequency and amount of pulse-synchronous blood pressure fluctuations. Accordingly, previous studies demonstrated that an elevated resting heart rate (RHR) was an independent risk factor for arterial stiffness [9]–[13]. We now put forward the hypothesis that in addition to pulse rate and independently of other cardiovascular risk factors, the product of pulse rate multiplied with blood pressure would be correlated with baPWV as a measure of arterial stiffness. We therefore conducted this study to examine the association between the blood pressure heart rate product and the baPWV in the asymptomatic polyvascular abnormalities community study (APAC).

## Methods

APAC is a community-based, observational, prospective, long-term follow-up study to investigate the epidemiology of asymptomatic polyvascular abnormalities in Chinese adults [14]. The APAC study was performed according to the guidelines of Helsinki Declaration and was approved by the Ethics Committees of the Kailuan General Hospital and Beijing Tiantan Hospital. Written informed consent was obtained from all participants. The study cohort was a sub-population of a previously described population of the Kailuan study which included a total of 101,510 employees and retirees (81,110 men) of the Kailuan (Group) Co. Ltd, a large coal mine industry located in Tangshan, Hebei Province. The city of Tangshan with approximately 7.2 million inhabitants in 2006 is situated 150 km southeast of Beijing and is a center of the coal mining industry. From June 2010 to June 2011, a sample of 7000 subjects older than 40 years was randomly selected from the Kailuan cohort, using a stratified random sampling method by age and gender based on the data of the Chinese National Census from 2010. A total of 5,852 subjects agreed to participate in the APAC study and 5,816 people eventually completed the baseline data collection. Among these 5,816 individuals, 376 subjects did not meet the following inclusion criteria (1) no history of stroke, transient ischemic attack, and coronary heart disease at baseline as assessed by a validated questionnaire; and (2) absence of neurologic deficits typical for stroke which as examined by experienced physicians. The group of these 376 individuals not included into the study as compared to the study participants did not differ significantly in age (*P* = 0.83) and gender (*P* = 0.79). A total of 5,440 participants were eventually eligible and participated in the APAC study.

BaPWV, blood pressure and RHR were assessed with the person sitting for at least five minutes in a controlled environment at a room temperature of 22°C to 25°C. The study participants had refrained from smoking and drinking of coffee, tea or alcohol for at least three hours nor had any exercise been performed for the last 30 minutes prior to the measurement of baPWV, blood pressure and RHR. All examinations were carried out by specially trained physicians and nurses. All subjects were in a supine position in light clothing without pillow and were asked to stop moving and talking at the time of the examination. Electrodes of the electrocardiogram were placed on both wrists, a microphone for detecting heart sounds was placed on the left edge of the sternum, and cuffs were wrapped on both arms and ankles. The lower edge of the arm cuff was placed 2–3 cm above the transverse striation of cubital fossa, while the lower edge of the ankle was placed 1–2 cm above the superior aspect of the medial malleolus. Two readings of baPWV were taken from each body side, and the second reading was used. Then, the higher value of the left side and right side was used for the data analyses. The rationale for this procedure was that the higher value represented the more severe arterial stenosis of the whole body. In addition, we also used the lower value of the left side and right side in the sensitivity analysis, and the results were similar.

Blood pressure was measured on the left arm to the nearest 2 mmHg using a mercury sphygmomanometer and standard procedures. Three readings of the systolic and diastolic blood pressure were recorded, taken at 5-minute intervals. The average of three readings was used for the statistical analysis. If two of the three measurements differed by more than 5 mmHg, an additional reading was taken. Mean arterial pressure (MAP) was calculated as diastolic blood pressure plus one third of the difference of systolic blood pressure minus diastolic blood pressure. A 10-second 12-lead electrocardiography was carried out to measure the RHR after the individual had rested in the supine position for 5 minutes. The number of R-R intervals (number of QRS complexes - 1) was divided by the time difference between the first and last heartbeat, and the results were converted to beats per minute (bpm).

All participants underwent a clinical examination and a standardized interview including questions on smoking and alcohol consumption, socioeconomic parameters, diet, lifestyle, family medical history, physical activity, and sleeping time and quality, known diagnosis of arterial hypertension, coronary heart disease, diabetes mellitus, hyperlipidemia, and stroke, and about the current treatment of these diseases. Anthropomorphic parameters such as body height and weight and waist und hip circumference were measured. The body mass index was calculated as the ratio of body weight (measured in kg) divided by the square of body height (measured in meter). Fasting blood samples were biochemically examined for the concentration of glucose, high-density lipoproteins (HDL), low-density lipoproteins (LDL), triglycerides, total cholesterol and high-sensitive C-reactive protein (CRP) [14], [15].

The statistical analysis was performed using a commercially available software program (SAS software (version 9.3; SAS Institute Inc., Cary, NC, USA).

Arterial stiffness was defined as baPWV≥1400 cm/s, since a baPWV≥1400 cm/sec has been described as an independent risk factor in the Framingham score and for the discrimination of patients with atherosclerotic cardiovascular disease [16]. We stratified the study population into quartiles of the product of RHR and systolic blood pressure (RHR-SBP) and into quartiles of the product of RHR and mean arterial blood pressure (RHR-MAP). We described continuous variables by their means ± standard deviations and categorical variables were described as percentages. We used the Student's t-test or ANOVA test for non-paired samples for the comparison of normally distributed parameters and the Wilcoxon or Kruskal-Waillis test for the comparison of non-parametric variables. The Chi-squared test was applied for the comparison of categorical variables. We performed multivariate logistic regression analyses to calculate odds ratios (OR) and 95% confidence intervals (CI) for the associations of the presence of arterial stiffness (defined as baPWV≥1400 cm/s) with RHR-SBP and RHR-MAP (the lowest quartile of the latter two variables was used as the reference category). Five models were used. Model 1 was the crude model. Model 2 included age and sex as independent variables. In model 3, we adjusted for age, sex, married status, educational level, reported income of each family member, body mass index, fasting blood concentrations of glucose, triglycerides, high-density lipoprotein cholesterol, low-density lipoprotein cholesterol, total cholesterol and high-sensitive C-reactive protein, current smoking status and current alcohol drinking status. In addition to the independent parameters analyzed in model 3, model 4/5 additionally included the RHR and the systolic blood pressure and the mean blood pressure in the list of independent parameters. Finally, we used multivariate linear regression to calculate the regression coefficient by treating baPWV, RSP and RMP as continuous variables in the model. The level for statistical significance was set at *P* = 0.05 in two-tailed tests.

## Results

Out of the 5,440 subjects who were originally included into the APAC study, we excluded 218 participants without measurements of baPWV and 69 individuals without RHR or blood pressure data. Eventually, 5153 (94.7%) subjects (3110 men) were eligible for inclusion in this study. Mean age was 55.1±11.8 years (range: 40–94 years).

**Figure 1:**
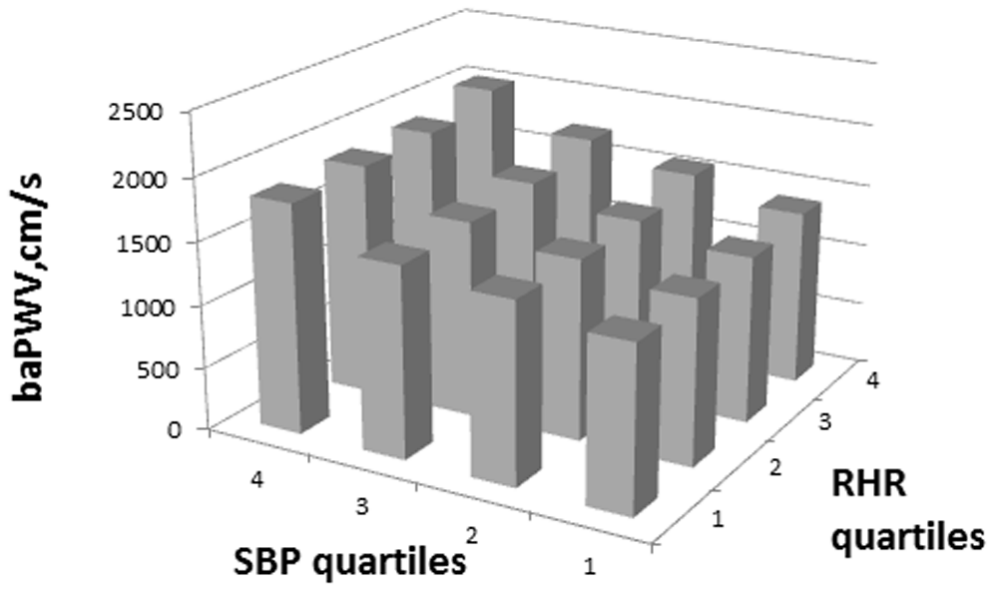
Integrated Resting Heart Rate Quartiles (RHR) and Systolic Blood Pressure (SBP) Quartiles and their Association with Brachial-Ankle Pulse Wave Velocity (baPWV).

**Figure 2:**
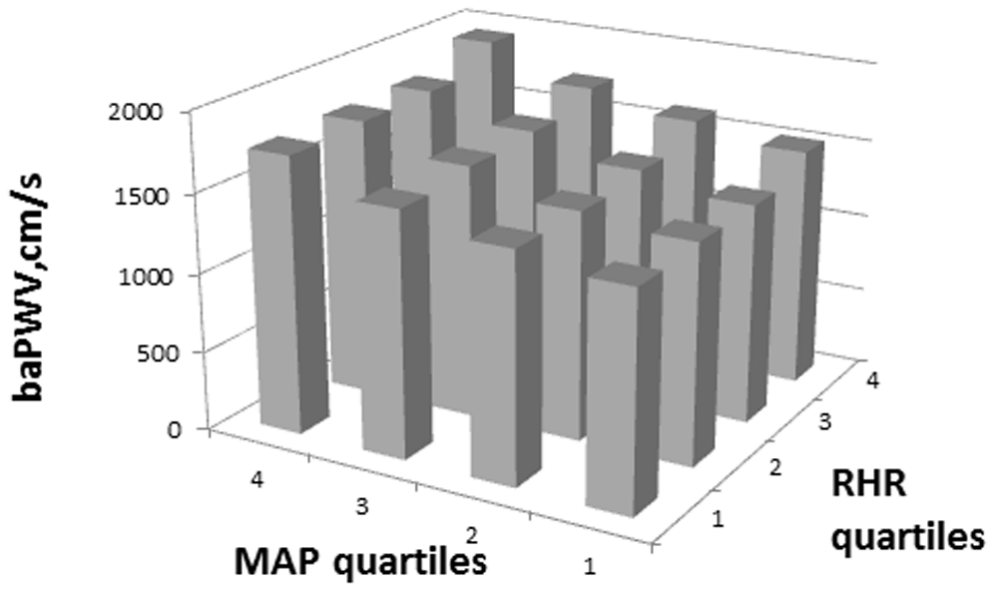
Integrated Resting Heart Rate Quartiles (RHR) and Mean Arterial Blood Pressure (MAP) Quartiles and their Association with Brachial-Ankle Pulse Wave Velocity (baPWV).

The mean baPWV was 1586±400 cm/s. Within each strata, significant (*P*<0.0001) linear relationships between the quartiles of all three variables, i.e., resting heart rate, mean arterial blood pressure or systolic blood pressure, and baPWV were found (Fig. 1, 2). The highest baPWV was observed in individuals from the highest quartiles of RHR and highest quartiles of systolic blood pressure or mean arterial blood pressure.

Out of the whole study population, 3190 (61.9%) individuals had a baPWV of ≥1400 cm/s. This subgroup with arterial stiffness compared to the subgroup with a baPWV of <1400 cm/s was significantly (*P*<0.05) older, had a higher proportion of men, higher body mass index, lower prevalence of married status and high-school graduation, higher reported income, higher prevalence of current smoking and alcohol consumption, higher systolic and diastolic blood pressure, higher blood concentration of glucose, low-density lipoprotein cholesterol, triglycerides, total cholesterol, and high-sensitive C-reactive protein, and lower concentrations of high-density lipoprotein cholesterol (Table 1).

**Table 1:** Comparison of Demographic and Other Characteristics of Participants with Arterial Stiffness (Brachial-Ankle Pulse Wave Velocity ≥1400 cm/s) and without Arterial Stiffness (Brachial-Ankle Pulse Wave Velocity <1400 cm/s) in the Asymptomatic Polyvascular Abnormalities Community Study.

	Brachial-Ankle Pulse Wave Velocity (cm/s)		*P*-Value
	<1400	≥1400	
n	1963	3190	
Age (Years)	48.12±6.39	59.42±12.32	<0.0001
Male Gender (Number (%))	945 (48.1%)	2165 (67.9%)	<0.0.001
Body Mass Index (kg/m^2^)	24.7±3.1	25.1±3.4	<0.0001
Marital Status (Married) (Number (%))	1939 (98.8%)	3082 (96.6%)	<0.0001
High-School Graduation (Number (%))	1865 (95.0%)	2654 (83.2%)	<0.0001
Reported Income of Each Family Member (≥1000¥) (Number (%))	1463 (74.5%)	2596 (81.4%)	<0.0001
Current Smoker (Number (%))	574 (29.2%)	1081 (33.9%)	0.0005
Current Alcohol Drinker (Number (%))	239 (12.2%)	501 (15.7%)	0.0004
Systolic Blood Pressure (mmHg)	119±14	139±19	<0.0001
Diastolic Blood Pressure (mmHg)	79±10	85±11	<0.0001
Mean Arterial Pressure (mmHg)	92.3±10.4	103±12	<0.0001
Resting heart rate (beats per minute)	68.3±9.2	71.8±11.8	<0.0001
Brachial-Ankle Pulse Wave Velocity (cm/s)	1250±100	1793±375	<0.0001
Resting Heart Rate Systolic Blood Pressure Product	8122±1532	9991±2232	<0.0001
Resting Heart Rate Mean Arterial Pressure Product	6317±1182	7418±1592	<0.0001
Fasting Blood Concentrations of:			
Glucose (mmol/L)	5.24±0.96	5.80±1.73	<0.0001
High-Density Lipoprotein Cholesterol (mmol/L)	1.67±0.47	1.60±0.44	<0.0001
Low-Density Lipoprotein Cholesterol (mmol/L)	2.53±0.66	2.69±0.83	<0.0001
Triglycerides (mmol/L)	1.50±1.25	1.79±1.51	<0.0001
Total Cholesterol (mmol/L)	1.43±0.64	1.60±0.73	<0.0001
High-Sensitive C-reactive Protein	1.52±2.83	2.53±4.82	<0.0001

The mean value of RHR-SBP was 9279±2191 and the mean value of RHR-MAP was 6999±1545. Participants with a higher RHR-SBP or a higher RHR-MAP were significantly older, had a higher proportion of men, higher body mass index, lower prevalence of high-school graduation, higher prevalence of current smoking and alcohol consumption, higher systolic and diastolic blood pressure, higher blood concentration of glucose, low-density lipoprotein cholesterol, triglycerides, total cholesterol, and high-sensitive C-reactive protein, and lower concentrations of high-density lipoprotein cholesterol (Tables 2, 3).

**Table 2:** Comparison of Demographic and Other Characteristics of the Participants of the Asymptomatic Polyvascular Abnormalities Community Study, Stratified by Resting Heart Rate Systolic Blood Pressure Product Quartiles.

	Resting Heart Rate Systolic Blood Pressure Product Quartiles				
	1	2	3	4	*P*-Value
n	1288	1288	1291	1286	
Age (Years)	52.22±10.58	53.89±11.15	55.55±12.00	58.81±12.44	<0.0001
Male Gender (Number (%))	679 (52.7%)	738 (57.3%)	845 (65.5%)	848 (65.9%)	<0.0001
Body Mass Index (kg/m^2^)	23.9±2.9	24.8±3.2	25.5±3.2	25.6±3.5	<0.0001
Marital Status (Married) (Number (%))	1263 (98.1%)	1260 (97.8%)	1260 (97.6%)	1238 (96.3%)	0.019
High-School Graduation (Number (%))	1185 (92.0%)	1146 (89.0%)	1129 (87.5%)	1059 (82.4%)	<0.0001
Reported Income of Each Family Member (≥1000 Chinese Yuan) (Number (%))	1021 (79.3%)	999 (77.6%)	1026 (79.5%)	1013 (78.8%)	0.64
Current Smoker (Number (%))	363 (28.2%)	381 (29.6%)	465 (36.0%)	446 (34.7%)	<0.0001
Current Alcohol Drinker (Number (%))	135 (10.5%)	177 (13.7%)	203 (15.7%)	225 (17.5%)	<0.0001
Systolic Blood Pressure (mmHg)	113±12	126±12	135±14	151±19	<0.0001
Diastolic Blood Pressure (mmHg)	75±8	81±9	85±9	90±11	<0.0001
Mean Arterial Pressure (mmHg)	87.7±8.8	95.9±8.5	101.9±9.2	110.4±11.7	<0.0001
Resting Heart Rate (Beats per Minute)	61.3±6.6	67.0±6.4	72.0±7.1	81.8±11.2	<0.0001
Brachial-Ankle Pulse Wave Velocity (cm/s)	1353±262	1488±308	1617±352	1887±446	<0.0001
Resting Heart Rate Systolic Blood Pressure Product	6884±640	8356±361	9642±425	12238±1741	<0.0001
Resting Heart Rate Mean Arterial Pressure Product	5343±562	6384±430	7290±528	8979±1286	<0.0001
Fasting Blood Concentrations of:					
Glucose (mmol/L)	5.22±1.08	5.44±1.36	5.62±1.47	6.07±1.89	<0.0001
High-Density Lipoprotein Cholesterol (mmol/L)	1.67±0.52	1.62±0.43	1.61±0.42	1.62±0.45	<0.0001
Low-Density Lipoprotein Cholesterol (mmol/L)	2.52±0.66	2.63±0.71	2.67±0.83	2.71±0.86	<0.0001
Triglycerides (mmol/L)	1.36±0.94	1.64±1.39	1.81±1.53	1.91±1.67	<0.0001
Total Cholesterol (mmol/L)	1.42±0.63	1.51±0.69	1.56±0.71	1.64±0.75	<0.0001
High-Sensitive C-reactive Protein	1.49±2.46	1.83±3.35	2.22±4.32	3.04±5.77	<0.0001

**Table 3:** Comparison of Demographic and Other Characteristics of the Participants of the Asymptomatic Polyvascular Abnormalities Community Study, Stratified by Resting Heart Rate Mean Arterial Pressure Product Quartiles.

	Resting Heart Rate Mean Arterial Pressure Product Quartiles				
	1	2	*3*	*4*	*P*-Value
n	1287	1289	1289	1288	
Age (Years)	54.57±12.33	54.34±11.47	55.15±11.58	56.41±11.77	<0.0001
Male Gender (Number (%))	686 (53.3%)	740 (57.4%)	818 (63.5%)	866 (67.2%)	<0.0001
Body Mass Index (kg/m^2^)	23.9±2.9	24.7±3.1	25.4±3.2	25.8±3.4	<0.0001
Marital Status (Married) (Number (%))	1255 (97.5%)	1253 (97.2%)	1259 (97.7%)	1254 (97.4%)	0. 89
High-School Graduation (Number (%))	1157 (89.9%)	1145 (88.8%)	1138 (88.3%)	1079 (83.8%)	<0.0001
Reported Income of Each Family Member (≥1000¥, in %) (Number (%))	1037 (80.6%)	1008 (78.2%)	1008 (78.2%)	1006 (78.1%)	0.34
Current Smoker (Number (%))	349 (27.1%)	382 (29.6%)	443 (34.4%)	481 (37.3%)	<0.0001
Current Alcohol Drinker (Number (%))	123 (9.6%)	170 (13.2%)	191 (14.8%)	256 (19.9%)	<0.0001
Systolic Blood Pressure (mmHg)	116±15	127±15	135±16	148±19	<0.0001
Diastolic Blood Pressure (mmHg)	73±8	80±8	85±8	92±10	<0.0001
Mean Arterial Pressure (mmHg)	88±9	96±8	102±9	111±11	<0.0001
Resting Heart Rate (Beats per Minute)	61±6	67±6	72±6	83±11	<0.0001
Brachial-Ankle Pulse Wave Velocity (cm/s)	1405±310	1504±333	1620±386	1815±438	<0.0001
Resting Heart Rate Systolic Blood Pressure Product	6983±780	839±656	9636±770	12101±1854	<0.0001
Resting Heart Rate Mean Arterial Pressure Product	5279±494	6358±247	7289±306	9067±1193	<0.0001
Fasting Blood Concentrations of:					
Glucose (mmol/L)	5.31±1.28	5.40±1.25	5.61±1.43	6.02±1.89	<0.0001
High-Density Lipoprotein Cholesterol (mmol/L)	1.67±0.53	1.64±0.44	1.61±0.42	1.60±0.44	0.002
Low-Density Lipoprotein Cholesterol (mmol/L)	2.51±0.67	2.62±0.70	2.67±0.84	2.72±0.84	<0.0001
Triglycerides (mmol/L)	1.37±0.98	1.58±1.26	1.80±1.58	1.96±1.71	<0.0001
Total Cholesterol (mmol/L)	1.43±0.64	1.52±0.69	1.57±0.71	1.62±0.75	<0.0001
High-Sensitive C-reactive Protein	1.59±2.48	1.90±3.83	2.20±4.20	2.89±5.57	<0.0001

In all five models of the multivariable logistic regression analysis, the OR for arterial stiffness (defined as baPWV≥1400 cm/s) increased significantly (*P*<0.0001) with increasing RHR-SBP quartile and higher RHR-MAP quartile. After adjusting for all independent variables assessed in the study and comparing the highest quartiles with the lowest quartiles of RHR-SBP or RHR-MAP revealed an OR of 2.72 (95%CI: 1.46–5.08) for RHR-SBP and an OR of 2.10 (95%CI: 1.18–3.72) for RHR-MAP (Table 4).

**Table 4:** Odds Ratios (95% Confidence Intervals) for the Associations between Arterial Stiffness (Defined as a Brachial-Ankle Pulse Wave Velocity ≥1400 cm/s) with Resting Heart Rate Systolic Blood Pressure Product Quartiles and Resting Heart Rate Mean Arterial Pressure Product Quartiles in the Asymptomatic Polyvascular Abnormalities Community Study.

	Quartile 1	Quartile 2	Quartile 3	Quartile 4
Resting Heart Rate Systolic Blood Pressure Product Range	4500–7737	7739–8998	9000–10488	10496–23114
Number of Participants with Arterial Stiffness (Number (%))	413 (13.0%)	714 (22.4%)	907 (28.4%)	1156 (36.2%)
Model 1	1	2.6 (2.2–3.1)	5.0 (4.2–5.9)	18.8 (15.2–23.4)
Model 2	1	3.0 (2.5–3.6)	5.6 (4.6–6.8)	19.0 (15.0–24.2)
Model 3	1	2.8 (2.3–3.4)	5.1 (4.1–6.2)	16.4 (12.7–21.0)
Model 4	1	1.5 (1.2–2.0)	1.8 (1.2–2.6)	2.7 (1.5–5.1)
Resting Heart Rate Mean Arterial Pressure Product, Range	2728–5908	5908–6784	6785–7855	7857–17921
Number of arterial stiffness (Number (%))	499 (15.6%)	704 (22.1%)	888 (27.8%)	1099 (34.5%)
Model 1	1	1.9 (1.6–2.2)	3.5 (3.0–4.1)	9.2 (7.6–11.1)
Model 2	1	2.6 (2.1–3.1)	5.2 (4.1–6.2)	13.9 (11.2–17.4)
Model 3	1	2.4 (2.0–3.0)	4.6 (3.7–5.6)	11.6 (9.2–14.7)
Model 5	1	1.3 (1.0–1.8)	1.6 (1.1–2.3)	2.1 (1.2–3.7)

Model 1: unadjusted.

Model 2: adjusted for age and sex.

Model 3: adjusted for age, sex, married status, educational level, reported income of each family member, body mass index, fasting blood concentrations of glucose, triglycerides, high-density lipoprotein cholesterol, low-density lipoprotein cholesterol, total cholesterol and high-sensitivity C-reactive protein, current smoking status, and current alcohol drinking status.

Model 4: adjusted for the same independent parameters as in Model 3, plus resting heart rate and systolic blood pressure.

Model 5: adjusted for the same independent parameters as in Model 3, plus resting heart rate and mean arterial pressure.

If instead the study population was not stratified into quartiles and the outcome parameters were not used as categorical variables but if baPWV, heart rate and blood pressure were taken as continuous variables, similar results in the statistical analysis were obtained. In all the multivariate linear regression analyses, elevated baPWV was significantly associated with increased RSP and RMP. RSP and RMP remained to be independently associated with baPWV after adjusting for all confounders (Table 5). In all these calculations, the variance inflation factor as part of the analysis of collinearity was lower than 10.

**Table 5:** Regression Coefficient of the Brachial-Ankle Pulse Wave Velocity with the Product of Resting Heart Rate and Blood Pressure.

	Resting Heart Rate Systolic Blood Pressure Product		Resting Heart Rate Mean Arterial Pressure Product	
	β	*P*-Value	β	*P*-Value
Model 1	0.0958	<0.0001	0.1069	<0.0001
Model 2	0.0744	<0.0001	0.0962	<0.0001
Model 3	0.0725	<0.0001	0.0928	<0.0001
Model 4 or 5	0.1047	<0.0001	0.0936	<0.0001

baPWV, Brachial-Ankle Pulse Wave Velocity.

Model 1: unadjusted.

Model 2: adjusted for age and sex.

Model 3: adjusted for age, sex, married status, educational level, reported income of each family member, body mass index, fasting blood concentrations of glucose, triglycerides, high-density lipoprotein cholesterol, low-density lipoprotein cholesterol, total cholesterol and high-sensitivity C-reactive protein, current smoking status, and current alcohol drinking status.

Model 4: adjusted for the same independent parameters as in Model 3, plus resting heart rate and systolic blood pressure.

Model 5: adjusted for the same independent parameters as in Model 3, plus resting heart rate and mean arterial pressure.

## Discussion

We tested the hypothesis that in addition to pulse rate and independently of other cardiovascular risk factors, the product of pulse rate multiplied with blood pressure would be correlated with baPWV as a measure of arterial stiffness. We found in our relatively large cross-sectional study, that a high product of resting heart rate multiplied with systolic blood pressure (RHR-SBP) and a high product of resting heart rate multiplied with the mean arterial blood pressure (RHR-MAP) were significantly associated with a higher prevalence of arterial stiffness. These associations were maintained after adjusting for systemic parameters in multivariate analysis. Fitting with the hypothesis, the findings suggest that RHR-SBP and RHR-MAP were independently of other systemic vascular risk factors associated with arterial stiffness.

The results of our study agree with the findings of previous investigations in that the baPWV was associated with factors such as older age, hypercholesterolemia, diabetes mellitus, cigarette smoking, higher blood concentration of C-reactive protein, and presence of a metabolic syndrome [17]–[29]. In addition to the previous findings, our data demonstrated a significant relationship between baPWV and RHR-SBP or RHR-MAP, independently of the factors the association of which with baPWV had previously been shown. The results of our study are in agreement with the reported association between a high heart rate and baPWV [9]–[11], as well as with the association between a high resting heart rate and an increased cardiovascular risk [30].

The results of our study may have importance clinically and in the discussion of the pathogenesis of arterial stiffening. Clinically, one may consider the product of heart rate and blood pressure as an additional marker in the assessment of the general vascular risk spectrum of patients. Pathogenetically, one has previously postulated that the age-related increase in pulse wave velocity is caused by a fatigue fracture of elastic elements in the arterial vessel wall [8]. The rate of elastin fracture may depend on the number of stress cycles and the amplitude of the related vessel wall stress [31]. Both parameters are measured by the pulse rate (i.e., frequency of stress cycles) and blood pressure. The results of our study that independently of other vascular risk factors the product of heart rate and blood pressure was associated with an increased baPWV in a general population, therefore supports the notion of arterial vessel wall related changes in dependence on the frequency of cyclic vessel wall movements and the amount of the movements.

Potential limitations of our study should be mentioned. First, a cross-sectional study as ours cannot prove a causal relationship which usually has to be shown in a longitudinal investigation. The results of our study only allow concluding on an association between RHR-SBP or RHR-MAP and arterial stiffness, while the role of RHR-SBP or RHR-MAP as a risk factor for arterial stiffness may be shown in follow-up investigations. The lack of analysis on causality may be of particular importance for our study, since it is unclear whether the observed association between baPWV and resting heart rate, blood pressure or the product of both was truly attributable to mechanical reasons, or only because they share some common risk factors, such as an increased sympathetic tone. Second, baPWV has limitations as a biomarker of arterial stiffness, since here is no real artery passing through the brachial point and ankle point. An alternative to the baPWVC would have been the carotid-femoral pulse wave velocity which may be addressed in future investigations. Third, the RHR was calculated from one electrocardiography recording. The RHR however depends on the daytime, so that the assessment of the mean RHR might have been more precise if performed in several measurements during a 24 hours profile.

In conclusion, a higher baPWV as a marker of arterial stiffness was associated with a higher product of RHR and blood pressure in multivariate analysis. In addition to other vascular risk factors, higher RHR in combination with higher blood pressure are risk factors for arterial stiffness. Using the product of RHR and blood pressure may be useful in the clinical assessment of the vascular risk spectrum of patients.
